# Primary lymphedema tarda

**DOI:** 10.11604/pamj.2014.19.16.4191

**Published:** 2014-09-08

**Authors:** Abdulrasheed Ibrahim

**Affiliations:** 1 Division of Plastic Surgery, Ahmadu Bello University Teaching Hospital Zaria Kaduna State Nigeria

**Keywords:** Primary lymphedema tarda, plastic surgery, Ulcerations

## Image in medicine

A 46 year old man presented to the plastic surgery clinic with a painless, progressive swelling of the right leg of seven years duration. He reported difficulty with ambulation and recurrent ulceration in the past three years. The patient's medical history was significant for hypertension. He had no allergies and no family history of lymphedema. On physical examination, the right leg was markedly enlarged from mid thigh to the foot. There were edematous changes with skin thickening. He had a septic ulcer on the medial surface of the leg (A). The mid leg circumference was 128 cm (B). Doppler findings of the leg were normal. Lymphedema tarda is a congenital disease characterized by underdevelopment of lymphatic pathways. It manifests commonly after the third decade as accumulation of lymph in the interstitial spaces of the skin. Wound healing is significantly impaired. Ulcerations which occur during the course of the disease heal slowly or not at all. The cause of this impairment is the continued lymphatic drainage as well as a regional oxygen deficiency in the diseased tissue. Wounds are managed by limiting infection and wound debridement. Due to the difficulty in managing wounds in lymphedema, prevention should be emphasized through rigorous skin care. Keeping the skin clean and dry as well as avoiding any form of trauma are essential components of prevention. Excisional surgery is indicated in ambulatory dysfunction and recurrent cellulitis. Surgery is usually successful, but can have a recurrence rate up to 50% over 10 years.

**Figure 1:**
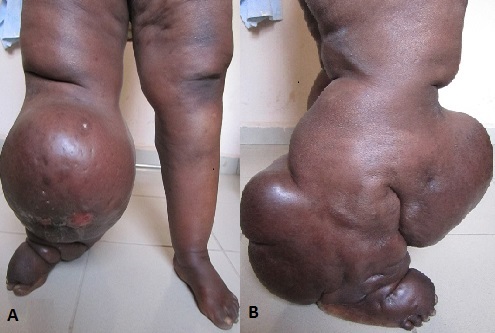
A) Patient's leg markedly enlarged from mid thigh to foot, anterior view; B) Lateral view of the involvement of the right leg

